# Uptake and impact of research for evidence-based practice: lessons from the Africa Health Systems Initiative Support to African Research Partnerships

**DOI:** 10.1186/1472-6963-14-S1-I1

**Published:** 2014-05-12

**Authors:** Adrijana Corluka, Marc Cohen, Esmé Lanktree, Renée Larocque

**Affiliations:** 1 Global Health Research Initiative, Ottawa, Canada

## Introduction

In 2008, the Global Health Research Initiative (GHRI) invited applications from teams of researchers and decision-makers who were interested in conducting research related to human resources for health and the implementation and use of integrated health information systems in Africa, with special attention to equity considerations. These thematic areas constituted the focus of the Africa Health Systems Initiative - Support to African Research Partnerships (AHSI-RES) program. GHRI is a partnership of three Canadian agencies: Foreign Affairs, Trade and Development Canada (DFATD), International Development Research Centre (IDRC), and the Canadian Institutes of Health Research (CIHR). It is hosted at IDRC.

AHSI-RES is a five year, $5.9 million CDN research program (2008-2014) supported by Foreign Affairs, Trade and Development Canada ($5 million) and the International Development Research Centre ($900 000). AHSI-RES is the research component of the larger Africa Health Systems Initiative (AHSI) program. The AHSI program is a 10 year, $450 million CDN commitment (2006-2016) to strengthening national-level health strategies and architecture, and is being implemented by Foreign Affairs, Trade and Development Canada.

The AHSI-RES program’s purpose is to support policy relevant research, knowledge translation and exchange in the program’s thematic areas. The AHSI-RES program emphasized the importance of ongoing interaction, collaboration, and exchange of ideas between researchers and decision-makers to maximize the likelihood that research findings would be used to inform programs and policies. A decision-maker was defined as ‘an individual who makes decisions about, or influences, health policies or practices.’

IDRC program officers engage with grantees in framing research problems, improving research designs, and choosing methodologies. IDRC staff and funded researchers work as peers to contribute new ideas and theories, influence practice and policy, and strengthen research networks, bringing together grantees to share research results [1]. This is known as the grants plus approach. Through this approach, program officers were able to respond to grantee-identified needs for building or strengthening local capacity for research, knowledge translation, and research use. The objective was to demonstrate a clear link between research, policy, and action to improve the health outcomes of the most vulnerable populations of the region.

Teams had to include one African researcher and one African decision-maker, both as co-principal applicants. The co-principal applicants had to be affiliated with an institution located in one of the AHSI-RES geographic areas of focus. Geographic areas of focus included: Francophone West Africa (Mali, Burkina Faso, Benin); Great Lakes and Eastern Africa (Tanzania, Uganda, Kenya); and, Southern Africa (Malawi, Mozambique, Zambia). Researchers and decision-makers affiliated with a non-African institution were eligible as co-applicants or collaborators, additional to the African co-principal applicants.

## Selection of research teams

Ten teams were selected from 57 proposals following a rigorous merit review process, where the review panel included international researchers and African decision-makers. Research teams that were successful in getting funding had to demonstrate: the ability to engage in interdisciplinary applied research to address complex policy-relevant questions related to the key themes of AHSI-RES; and, the ability to link research, policy, and action to improve health decision-making and programming. Gender, ethics, capacity development, and knowledge translation and exchange were cross-cutting themes that featured in AHSI-RES programming.

Research teams were based in: Burkina Faso, Mali, Kenya, Tanzania, Uganda, Malawi, and Zambia, and some involved Canadian researchers. Their research focused on two main areas: 1) the recruitment and retention of health workers and the shifting of certain services to less specialized health workers in response to a severe human resources crisis in the health sector in sub-Saharan Africa; and 2) the role of health information in ensuring greater equity in access to health care. The results of AHSI-RES research on the recruitment and retention of health workers is presented in a supplement to *Human Resources for Health.* This supplement focuses on lessons learned from the uptake and impact of research for evidence-based practice.

## Health Systems Research: focus on research for evidence-based policies and practice

Health systems research can enrich the evidence base needed to inform the development and implementation of equity-oriented policies and interventions. The expected outcomes for AHSI-RES include: (i) improved and timely uptake of research evidence for health systems strengthening among decision makers in low- and middle-income countries; (ii) improved knowledge base for health systems strengthening that addresses the needs of vulnerable populations, focusing on priority global health issues in low- and middle-income countries; and (iii) strengthened individual and institutional capacity for doing and using research on health systems strengthening in low- and middle-income countries.

## Policy-linked results and lessons learned

The articles in this supplement present research results from Kenya, Malawi, Mali, Uganda, Tanzania, and Zambia**.** The research ranges from investigation into government policies to retain health workers in underserved areas, to the implementation and validation of health information systems in peri-urban and rural areas.

Findings from these research teams capture the complexity of challenges faced by decision-makers, health system managers, and health human resources in establishing, maintaining, and sustaining effective and functional health systems. The research serves to test, validate, and challenge policies at national and regional levels. Some of the policies and strategies incorporated in this supplement’s research include, amongst others: Kenya’s Community Health Strategy, Uganda’s integrated Community Case Management (iCCM) strategy, as well as the World Health Organization and International Agency for Prevention of Blindness’s program strategy for “VISION 2020: The Right to Sight,” a global initiative to eliminate avoidable blindness.

For example, as described in the articles, the policy-relevant results and lessons learned include:

• the Kenya Community Health Strategy is an effective approach to delivering community-based interventions: it can be successfully implemented and sustained in different socio-demographic contexts, with participatory community planning, based on household information, driving the improvement of health indicators. There were significant changes in essential maternal and newborn care practices such as antenatal care attendance and skilled deliveries because household members had been provided with the necessary information to make healthy decisions to be able to respond to maternal and neonatal health needs, based on evidence provided by the community based health information system [2].

• a better understanding of the quality of eye care at primary health care facilities and influencing factors (e.g. staff turnover and absenteeism) in Kenya, Malawi and Tanzania, are urgently needed before continuing to invest resources in the scale up of the model of task shifting in Africa as it relates to VISION 2020: The Right to Sight, a global initiative to eliminate avoidable blindness. This study demonstrated the negative impact of the high turnover of primary health care workers and absenteeism in providing health services at the community level. This turnover and absenteeism also affects the integrity of research on interventions designed to improve health services [3].

• in Uganda, specific iCCM training and support to a selected segment of community health workers (CHWs) within larger CHW village teams unexpectedly and negatively impacts ‘basic’ CHWs’ work motivation, but not necessarily team functioning. The Ugandan Ministry of Health’s national strategy calls for the employment of volunteer CHWs for the implementation of iCCM of common illnesses (diarrhea, acute respiratory infection, pneumonia, fever/malaria) affecting children under five years of age. The researchers recommend that CHW programmers should consider minimizing segregation when introducing new program opportunities through providing equal opportunities to participate and receive incentives, while seeking means to improve communication, CHW solidarity, and motivation [4].

## Conclusion

The 10 teams supported by the AHSI-RES program worked to connect research with policy and action to improve health decision-making and programming in the sub-Saharan region, paying particular attention to the needs of disadvantaged segments of the population. Grantees benefited from opportunities to strengthen individual and institutional capacity for doing and using research on health systems strengthening. Research teams also benefitted from linking with African decision-makers to make the research collaborative and responsive, enhancing the likelihood of research uptake to inform current and future policies.

## Competing interests

Adrijana Corluka and Renée Larocque serve as Senior Program Officers, Marc Cohen serves as Program Officer, and Esmé Lanktree serves as Program Management Officer, with the Global Health Research Initiative. The Global Health Research Initiative supported the assembly and publication of this supplement. The views expressed in this introductory article are those of the authors alone and do not represent the views of the Global Health Research Initiative, the International Development Centre, the Canadian Institutes of Health Research, nor Foreign Affairs, Trade and Development Canada.

## Declarations

The publication costs associated with this article are funded by Foreign Affairs, Trade and Development Canada and the International Development Research Centre through the Global Health Research Initiative.

This article has been published as part of *BMC Health Services Research* Volume 14 Supplement 1, 2014: Uptake and impact of research for evidence-based practice: lessons from the Africa Health Systems Initiative's research component. The full contents of the supplement are available online at http://www.biomedcentral.com/bmchealthservres/supplements/14/S1

## References

[B1] International Development Research Centre Who can apply? Developing country researchers http://www.idrc.ca/en/funding/whocanapply/pages/researcherslist.aspx

[B2] Olayo R Wafula C Aseyo E Loum C Kaseje DCO A quasi-experimental assessment of the effectiveness of community health strategy on health outcomes in Kenya BMC Health Services Research 2014 14 Suppl 1 S5 10.1186/1472-6963-14-S1-S3 PMC4108865 25079378

[B3] Kalua K Gichangi M Barassa E Eliah E Lewallen S Courtright P A randomised controlled trial to investigate effects of enhanced supervision on primary eye care services at health centres in Kenya, Malawi and Tanzania BMC Health Services Research 2014 14 Suppl 1 S6 10.1186/1472-6963-14-S1-S6 PMC4108898 25079942

[B4] Mercader HFG Kyomuhangi T Buchner DL Kabakyenga J Brenner JL Drugs for some but not all: Inequity within community health worker teams during introduction of integrated community case management BMC Health Services Research 2014 14 Suppl 1 S1 10.1186/1472-6963-14-S1-S1 PMC4108853 25078968

